# Effect of remimazolam besylate compared with propofol on the incidence of delirium after cardiac surgery: study protocol for a randomized trial

**DOI:** 10.1186/s13063-021-05691-x

**Published:** 2021-10-18

**Authors:** Maopeng Yang, Xinyan Liu, Daqiang Yang, Yahu Bai, Bingxin Qin, Shoucheng Tian, Ranran Dong, Xuan Song

**Affiliations:** 1 Liaocheng Cardiac Hospital, Shandong, 252200 China; 2 Dong E Hospital Affiliated to Shandong First Medical University, Shandong, 252200 China; 3 ICU, Shandong First Medical University, Shandong, 250117 China; 4 ICU, Shandong Provincial Hospital Affiliated to Shandong First Medical University, Shandong, 250021 China

**Keywords:** Remimazolam besylate, Propofol, Cardiac surgery, Delirium

## Abstract

**Background:**

Delirium is an acute cognitive disorder that presents with fluctuation in cognition, apathy, and non-organized thinking, resulting in increased morbidity, mortality, intensive care unit (ICU) stay, and total healthcare costs. In patients undergoing cardiac surgery, delirium also increases the risk of postoperative complications, such as respiratory insufficiency, sternum instability, and need for re-operation of the sternum. This study aims to understand the incidence of delirium in patients after cardiac surgery in patients sedated with remimazolam besylate versus propofol.

**Methods:**

In this prospective, double-blind, randomized controlled clinical trial, we aim to recruit 200 patients undergoing cardiac surgery between January 1, 2021, and December 31, 2021, who will be randomized to receive either remimazolam besylate or propofol infusions postoperatively, until they are extubated. The primary outcome is the incidence of delirium within 5 days after surgery. Secondary outcomes include the time of delirium onset, duration of delirium, ICU length of stay, hospital length of stay, and mechanical ventilation time.

**Discussion:**

The key objective of this study is to assess whether remimazolam besylate reduces the incidence of delirium in patients after cardiac surgery compared to propofol sedation. In this preliminary randomized controlled clinical trial, we will test the hypothesis that the use of remimazolam besylate lowers the incidence of delirium when compared to propofol in patients undergoing cardiac surgery.

**Trial registration:**

chictr.org.cn ChiCTR2000038976. Registered on October 11, 2020

## Background

Delirium is an acute brain illness that alters consciousness, attention, cognition, and perception [1]. According to a systematic review, postoperative delirium (POD) occurs in 11 to 51% of patients after surgery and is increasingly prevalent with age [2]. Delirium is associated with increased morbidity and mortality, prolonged hospital stay, worse functional recovery, and a long-term decline in cognitive function [2, 3]. Among hospitalized patients, around 30 to 40% of delirium cases are thought to be attributable to modifiable risk factors, such as insufficient sedation and analgesia, and are therefore preventable [4]. Pain is associated with an acute stress response, including changes in heart rate, blood pressure, respiratory rate, neuro-endocrine secretion, and psychological distress, such as agitation [5, 6].

The current anesthesia practice for sedating patients during a procedure involves the use of two major hypnotics, midazolam, and propofol, often in combination with an opioid analgesic, typically fentanyl, or sufentanil. Propofol is a potent intravenous sedative/hypnotic agent with a 15- to 40-s onset of action and an extremely short half-life [7, 8], allowing for rapid recovery. These properties make propofol an appealing agent for procedural sedation either alone or in combination with an opioid [9–13]; however, the disadvantage is potential respiratory depression or hypotension without a reversal agent [14], requiring constant monitoring by an anesthesia provider. Furthermore, propofol use has been associated with delirium in some, but not all, clinical studies [15]. This delirium may be the result of the fact that propofol has been shown to interact with muscarinic acetylcholine receptors. Medications targeting these receptors have previously been shown to be associated with delirium [15].

In contrast, remimazolam is a new short-acting gamma-aminobutyric acid (GABA) (A) receptor agonist, and pharmacokinetic and pharmacodynamic modeling of arterial remimazolam concentration and effect measures can be achieved with either mamillary or recirculatory models [16–19]. Remimazolam has an onset of action of 1 to 3 min [18] and a terminal half-life of 0.75 h and is metabolized by liver-bound carboxylesterase-1 to an inactive metabolite. Unlike propofol, remimazolam undergoes organ-independent metabolism by tissue esterases into an inactive metabolite. The extent and duration of sedation are dose-dependent, and remimazolam is eliminated by first-order pharmacokinetics, with no clear relationship between body weight and elimination clearance. Consequently, prolonged infusions or higher doses are unlikely to result in accumulation and extended effect, making it favorable for use as an intravenous anesthetic [20]. In the case of remimazolam as a perioperative hypnotic, similar to other benzodiazepines, it may be associated with delirium. However, its rapid elimination might offer some protection [21]. Future research is necessary to clarify the role that remimazolam may have in causing delirium [21].

Remimazolam besylate has been approved by the National Medical Products Administration as a new drug for use during anesthesia and sedation in 2019 [22]. Similar to remimazolam, remimazolam besylate has a short half-life, resulting in quick-acting onset and recovery compared with currently available short-acting sedatives [23, 24]. However, the sedative effects and safety of remimazolam in the context of delirium have not been studied. This trial aims to evaluate the incidence of delirium with the use of remimazolam besylate versus propofol in patients undergoing cardiac surgery.

## Methods

### Study setting

This prospective, double-blind randomized controlled study will take place in the surgical ICU of Liaocheng Cardiac Hospital in Shandong Province, China, upon obtaining approval from the ethics committee and written informed patient consent is obtained.

### Participant characteristics

**Fig. 1:**
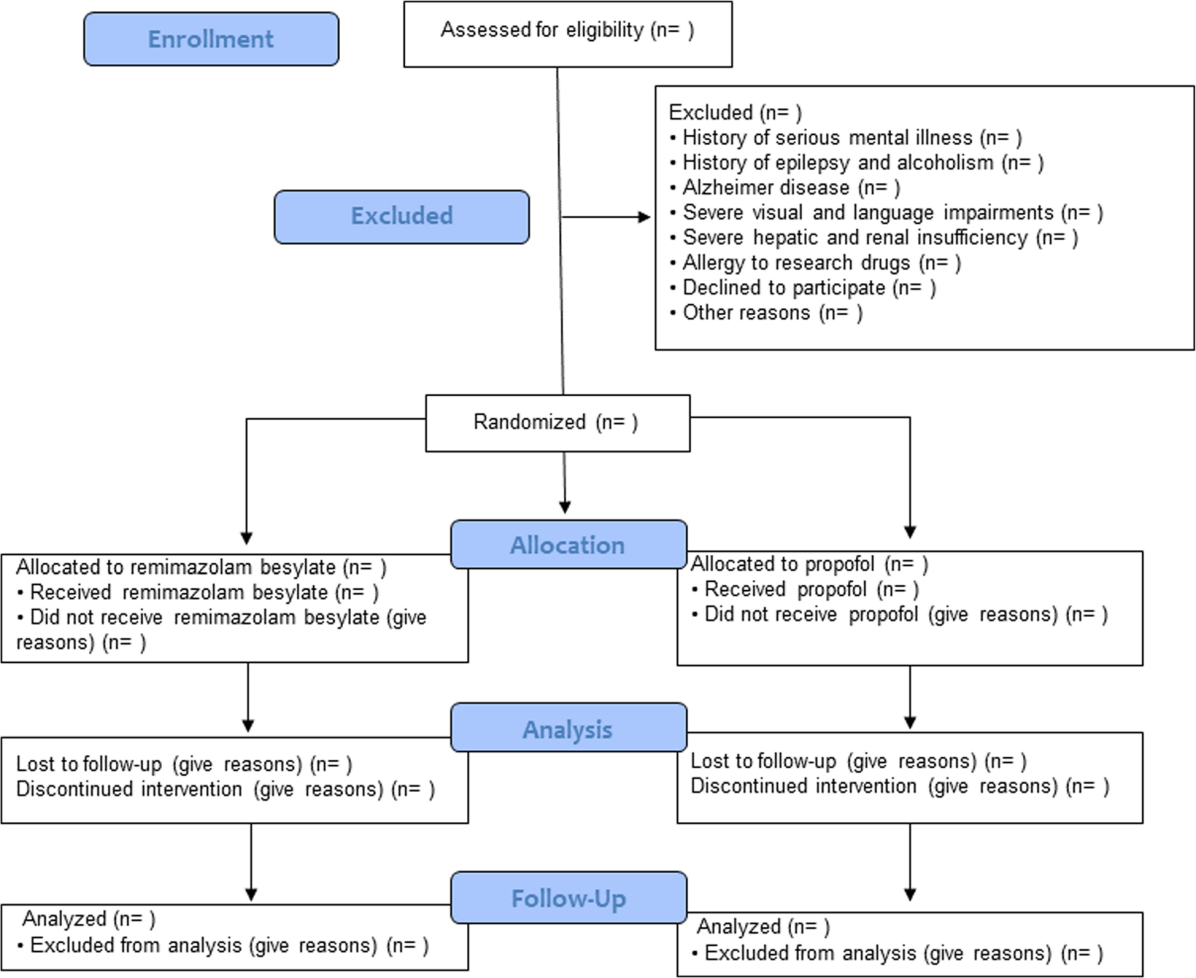
Simplified schematic of the trial design

Patients will be included if they are ≥18 years old, are scheduled to undergo coronary artery bypass surgery or valve replacement surgery, and are admitted to the surgical ICU between January 1, 2021, and December 31, 2021. Patients will be excluded if they have a history of serious mental illness; a recent history of epilepsy and/or alcoholism; a history of Alzheimer’s disease, Parkinson’s disease, or severe visual or language impairments, impacting their ability to communicate; severe hepatic and renal insufficiency; or if they are allergic to research drugs. A simplified schematic of the trial design is shown in Fig. 1.

### Anesthesia and cardiopulmonary bypass (CPB) management

Anesthesia management is standardized to minimize the impact of the type of anesthetic on neurological outcomes. Anesthesia will be induced with 0.3 mg/kg etomidate, 0.3 mg/kg cis-atracurium, 2–3 μg/kg sufentanil, and 0.05 mg/kg midazolam and maintained with 1.0% seven halothane, 150 mg/h propofol, and 0.5 μg/kg/h dexmedetomidine; 0.1 μg/kg sufentanil can be added when the analgesic effect is weakened, with a target bispectral index (BIS) between 40 and 60. The heart rate and blood pressure will be maintained within 25.0% of baseline values. Anticoagulation will be achieved with heparin to maintain an activated clotting time above 480 s. The same type of ultrafilter (Dideco) will be used in patients needing cardiopulmonary bypass (CPB). Management of CPB will include systemic temperature drift to 34°C, targeted mean perfusion pressure between 60 and 80 mm Hg, and pump flow rates of 2.0 to 2.4 l/min/m^2^. A deep hypothermic circulatory arrest will be achieved by cooling to 20°C with antegrade cerebral perfusion. Before separation from CPB, patients will be rewarmed to 36 to 37°C. After separation from CPB, heparin will be neutralized with protamine sulfate (1 mg/100 U heparin) to achieve an activated clotting time within 10% of baseline. All patients will be transferred to the surgical ICU after surgery.

### Study drug administration

Patients are randomly allocated to either the remimazolam besylate group (R group) or the propofol group (P group) according to a computer-generated randomization code in blocks of four, aiming at subject allocation in a 1:1 ratio. Opaque-sealed envelopes are generated according to the randomization schedule and will be opened by a study coordinator before surgery. After surgery, patients in both groups will receive sufentanil infusion of 0.2 to 0.3 μg/kg/h. R group patients will receive remimazolam besylate infusion at 0.25 to 1 mg/kg/h, and P group patients will receive propofol infusion at 1 to 4 mg/kg/h until they are ready for tracheal extubation. Patients and outcome assessors will be blinded to their assignment.

### Analgesia, sedation, and delirium evaluation

Analgesia and sedation assessments will be performed every 4 h after the surgery. The level of sedation will be assessed using the Richmond Agitation-Sedation Scale (RASS, Table 1) to keep the patient in a lightly sedated state (calm and cooperative, RASS-1 to RASS-2). The Critical-Care Pain Observation Tool (CPOT, Table 2) will be used for pain assessment during mechanical ventilation, with the control target of CPOT < 3. After the removal of tracheal intubation, the Numeric Rating Scales (NRS) will be used and the control target is NRS < 3. If pain control fails to achieve the goal, an additional dose of sufentanil will be given. Delirium will be assessed using the Intensive Care Delirium Screening Checklist (ICDSC, Table 3), every 12 h for 5 days after surgery. The ICDSC scale includes the evaluation of 8 delirium-related characteristics, including consciousness, attention, orientation, hallucinations, psychomotor excitement or retardation, sleep cycle, language, and symptom fluctuations. Each item is given 1 point, and the total score is 8 points, such that ICDSC ≥4 is considered to be delirium, 1–3 is subdelirium syndrome, and 0 is no delirium. The diagnosis of delirium will be made by experienced clinicians.

**Table 1:** Richmond Agitation-Sedation Scale (RASS)

Score	Term	Description
+4	Combative	Overtly combative or violent; immediate danger to staff
+3	Very agitated	Pulls on or removes tube(s) or catheter(s), or has aggressive behavior toward staff
+2	Agitated	Frequent non-purposeful movement or patient-ventilator dyssynchrony
+1	Restless	Anxious or apprehensive but movements not aggressive or vigorous
0	Alert and calm	
−1	Drowsy	Not fully alert, but has sustained (>10 seconds) awakening with eye contact to voice
−2	Light sedation	Briefly (<10 seconds) awakens with eye contact to voice
−3	Moderate sedation	Any movement (but no eye contact) to voice
−4	Deep sedation	No response to voice, but any movement to physical stimulation
−5	Unarousable	No response to voice or physical stimulation

**Table 2:** The Critical-Care Pain Observation Tool (CPOT)

Indicator	Description	Score (total score 0–8)
**Facial expressions**	No muscle tension observed	Relaxed, neutral 0
	Presence of frowning, brow lowering, orbit tightening, and levator contraction or any other change (e.g., opening eyes or tearing during nociceptive procedures)	Tense **1**
	All previous facial movements plus eyelid tightly closed (the patient may present with mouth open or biting the endotracheal tube)	Grimacing **2**
**Body movements**	Does not move at all (does not necessarily mean absence of pain) or normal position (movements not aimed toward the pain site or not made for the purpose of protection)	Absence of movements or normal position **0**
	Slow, cautious movements, touching or rubbing the pain site, seeking attention through movements	Protection **1**
	Pulling tube, attempting to sit up, moving limbs/thrashing, not following commands, striking at staff, trying to climb out of bed	Restlessness/agitation **2**
**Muscle tension**	No resistance to passive movements	Relaxed **0**
	Resistance to passive movements	Tense, rigid **1**
	Strong resistance to passive movements or incapacity to complete them	Very tense or rigid **2**
**Compliance with the ventilator** (intubated patients) **OR**	Alarms not activated, easy ventilation	Tolerating ventilator or movement **0**
	Coughing, alarms may be activated but stop spontaneously	Coughing but tolerating **1**
	Asynchrony: blocking ventilation, alarms frequently activated	Fighting ventilator **2**
**Vocalization** (extubated patients)	Talking in normal tone or no sound	Talking in normal tone or no sound **0**
	Sighing, moaning	Sighing, moaning **1**
	Crying out, sobbing	Crying out, sobbing **2**

**Table 3.** The Intensive Care Delirium Screening Checklist

Patient evaluation	Score (total score 0–8)
**Altered level of consciousness (A–E)**	
If A or B do not complete patient evaluation for the period)	
A: No response	0
B: Response to intense and repeated stimulation (loud voice and pain)	0
C: Response to mild or moderate stimulation	1
D: Normal wakefulness	0
E: Exaggerated response to normal stimulation	1
**Inattentation**	
Difficulty in following a conversation or instructions.	0 or 1
Easily distracted by external stimuli.	
Difficulty in shifting focuses.	
Any of these scores 1 point.	
**Disorientation**	
Any obvious mistake in time, place, or person scores 1 point.	0 or 1
**Hallucination-delusion-psychosis**	
The unequivocal clinical manifestation of hallucination or of behavior probably due to hallucination or delusion.	0 or 1
Gross impairment in reality testing.	
Any of these scores 1 point.	
**Psychomotor agitation or retardation**	
Hyperactivity requiring the use of additional sedative drugs or restraints in order to contral potential danger to oneself or others.	0 or 1
Hypoactivity or clinically noticeable psychomotor slowing.	
Any of these scores 1 point.	
**Inappropriate speech or mood**	
Inappropriate, disorganized, or incoherent speech.	0 or 1
Inappropriate display of emotion related to events or situation.	
Any of these scores 1 point.	
**Sleep/wake cycle disturbance**	
Sleeping less than 4h or waking frequently at night (do not consider wakefulness initiated by medical staff or loud environment).	0 or 1
Sleeping during most of the day.	
Any of these scores 1 point.	
**Symptom fluctuation**	
Fluctuation of the manifestation of any item or symptom over 24h scores 1 point.	0 or 1

### The treatment of delirium

The management of delirium is standardized. Patients with ICDSC ≥4 will be treated with haloperidol combined with dexmedetomidine. First, patients will receive a bolus of 5 to 10 mg haloperidol, and the administration would be repeated every 30 to 60 min until the patient is quiet. Subsequently, patients will receive dexmedetomidine infusion 0.2–0.7 μg/kg/h as needed.

### Data collection

The following data will be collected: patient demographic information, preoperative medications (calcium channel blocker [CCB], angiotensin-converting enzyme inhibitor/angiotensin receptor blocker [ACEI/ARB], beta-receptor blockers, statins, antiplatelet drugs, benzodiazepines, etc.), preoperative New York Heart Association [NYHA] grading, left ventricular ejection fraction [LVEF, %], type of surgery (coronary artery bypass grafting [CABG], valve replacement), operation time, extracorporeal circulation time, aortic block time, intraoperative hypotension (systolic pressure<90 mm Hg, duration ≥5 min) and its duration, baseline of hemoglobin and creatinine, postoperative conditions and complications (pacemaker, intra-aortic balloon pump [IABP], type of vasoactive drugs, re-exploration for bleeding, arrhythmia, stroke, sepsis, hemofiltration, hypotension and its duration), the dosage of propofol and remimazolam besylate, analgesia and sedation scores and ICDSC scores every 4 h within 5 days after surgery, mechanical ventilation time (from return to ICU after surgery to successful weaning), ICU length of stay, and hospital length of stay (the number of days in hospital after surgery). Before the initiation of the study, an electronic case report form will be established with password-protected access. Each enrolled patient will be assigned an identification number. Patient data will be coded and kept confidential. We will indicate a coordinator to ensure the integrity of data collection and timely completion of the case report form. Trial conduct will be audited by persons independent from investigators with no competing interests every 3 months.

### Outcome measures

The primary outcome is the incidence of delirium (ICDSC ≥ 4) within 5 days after surgery. Secondary outcomes include the time of delirium onset (the first time of ICDSC ≥ 4 within 5 days after surgery), the duration of delirium (the duration of ICDSC ≥ 4), ICU length of stay, hospital length of stay (the number of days hospitalized after surgery), and mechanical ventilation time (from admitting to surgical ICU post-surgery to successful weaning).

### Adverse events

Adverse events will be monitored during the procedure. Investigators will record all adverse events, including severe hypotension and respiratory depression. If the above adverse events occur, bedside physicians may suspend study participation and unblind the participant. In such cases, the events leading to study suspension will be recorded and reported to the Institutional Review Board of Liaocheng Cardiac Hospital within 24 h.

### Sample size and statistical analysis

Based on previous studies, we estimated the incidence of delirium during sedation with propofol after cardiac surgery as 31.5%, *α*=0.05, and the statistical power of 0.8. The sample size was estimated by comparing the rates of two independent samples. Each group will need to include 91 patients. A two-tailed Student’s *t* test will be used for two independent samples to analyze continuous normally distributed data. The Mann-Whitney *U* test will be applied for nonparametric data. Our per-protocol analysis will exclude patients who deviated from the protocol. Therefore, our study may suffer from attrition bias, in which the groups of patients being compared no longer have similar characteristics. For the primary outcome of delirium, the two groups will be compared using the chi-square test. Relative risk and confidence intervals (CIs) for proportions will be calculated at 95%. *P* ≤0.05 will be considered statistically significant. Statistical analysis will be performed using SPSS 20.0 software (SPSS Software, Chicago, IL, USA).

### Ethics and dissemination

The study protocol was approved by the Institutional Review Board of Liaocheng Cardiac Hospital (CN-2020032). The trial was registered at chictr.org.cn with identifier ChiCTR2000038976 on October 11, 2020. The results of the study will be presented at relevant national and international conferences and submitted to international peer-reviewed journals. There are no plans to communicate results specifically to participants.

## Discussion

The current study will be the largest prospective randomized clinical trial evaluating the possibility of using remimazolam besylate-based operative sedation to reduce delirium in patients after cardiac surgery compared with propofol. Although several recent studies have compared the safety and sedation efficacy of remimazolam and propofol [25–27], none of them have focused specifically on delirium. Propofol use has been associated with delirium in some, but not all, clinical studies [15]. In the case of remimazolam as a perioperative hypnotic, further research is necessary to clarify the role that remimazolam may have in causing delirium [21].

Postoperative delirium is a serious and often under-recognized complication, characterized by altered consciousness, changes in cognitive abilities, and rapid onset that results in an increase in length of hospital stay and poor outcomes [28–30]. In patients undergoing CABG, the occurrence of Parkinson’s disease is associated with a higher rate of postoperative stroke and mortality [31]. If a patient suffers from hyperactive delirium, complications such as self-extubation, the exit of life-saving catheters, and asynchrony between patient and ventilator are increased, which portends poor prognosis [32]. Since efficacious treatment options are lacking, efforts should be made to prevent delirium [33]. To adequately prevent delirium, reversible factors, including pain and anemia, should be minimized and an appropriate therapy should be established.

Postoperative sedation practices have evolved, targeting a more balanced regimen of hypnotic- and analgesia-based sedation. Sedation approaches are generally based on either midazolam or propofol and often vary widely across the globe [34, 35]. Recently, dexmedetomidine is considered as an attractive, alternative sedative, with many studies demonstrating the relationship between dexmedetomidine and reduced delirium after cardiac surgery [32, 36]. A meta-analysis of 14 prospective randomized clinical trials found that sedation with dexmedetomidine reduced delirium rates in critically ill patients compared to sedation with midazolam [37]. A Cochrane review, however, found that there was no evidence for a beneficial effect of dexmedetomidine on the risk of delirium in critically ill patients due to the high heterogeneity of the studies, inadequate assessment of delirium, and lack of having delirium as a primary outcome measure [38]. Additionally, dexmedetomidine is often suitable for mild sedation, and it is difficult to achieve the goal of sedation with dexmedetomidine alone.

The primary benefits of remimazolam besylate include short duration of action, limited accumulation, minimal risk of respiratory depression, minimal perturbation of heart rate and blood pressure, and availability of a reversal agent, making this drug widely marketable for sedation by both anesthesiologists and non-anesthesiologists. Remimazolam besylate presents an attractive alternative in elderly patients after surgery; however, its association with delirium has not been studied. The purpose of this study is to evaluate whether remimazolam besylate can reduce the incidence of delirium when compared to propofol in patients undergoing cardiac surgery.

### Strengths and limitations

To our knowledge, this is the first study to evaluate whether remimazolam besylate can reduce the incidence of delirium in patients after cardiac surgery. This prospective, double-blind randomized controlled clinical trial has been carefully designed and will be meticulously implemented. However, there are some limitations to our research. Most notably, all participants will be screened and enrolled after ICU admission and will not have baseline delirium assessment with cognitive function assessment; therefore, we cannot preclude the potential bias introduced by a preoperative imbalance of baseline conditions.

### Trial status

At the time of manuscript submission, the study is in the preparation phase for recruitment. This is the first version of the protocol completed on September 30, 2020. Recruitment is scheduled to begin in January 2021 and expected to be completed by December 2021.

## Data Availability

The dataset(s) supporting the conclusions of this article, model consent form, and other related materials will be available by the corresponding author’s email.

## Abbreviations

POD: Postoperative delirium
ICU: Intensive care unit
BIS: Bispectral index
CPB: Cardiopulmonary bypass
RASS: Richmond Agitation-Sedation Scale
ICDSC: Intensive Care Delirium Screening Checklist
CPOT: Critical care Pain Observation Tool
GABA: Gamma-aminobutyric acid
NRS: Numeric Rating Scales
ICDSC: Intensive Care Delirium Screening Checklist
CCB: Calcium channel blocker
ACEI/ARB: Angiotensin-converting enzyme inhibitor/angiotensin receptor blocker
NYHA: New York Heart Association
LVEF: Left ventricular ejection fraction
CABG: Coronary artery bypass grafting
IABP: Intra-aortic balloon pump
Cis: Confidence intervals
